# Retinal ischemia after cardiovascular interventions: Neuroimaging correlates and timing phenotypes in a consecutive series

**DOI:** 10.1007/s00417-026-07143-1

**Published:** 2026-02-24

**Authors:** Or Gil, Avner Hostovsky, Noah Benjamin Nagar, Shalev Fried, Iris Moroz, Gabriel Katz, Ari Leshno, Miri Fogel Levin

**Affiliations:** 1 Retina clinic, Goldschleger Eye Institute, Sheba Medical Center, Tel Hashomer, Ramat Gan, Israel; 2 Tel Aviv University, Tel Aviv, Israel

**Keywords:** Retinal ischemia, Retinal artery occlusion, Paracentral acute middle maculopathy, Cardiovascular procedures, Neuroimaging, Embolism

## Abstract

**Purpose:**

To characterize symptomatic retinal ischemic events following cardiovascular procedures and evaluate their clinical features and association with cerebral ischemia.

**Methods:**

We conducted a retrospective review of OCT-confirmed acute retinal ischemia at a tertiary center (2015–2024), including patients who had undergone a cardiovascular procedure within 30 days preceding symptom onset.

**Results:**

Thirteen patients (mean age 67±8.5 years, 84.6% male) developed retinal ischemic events following cardiac catheterization (n=4), valve replacement (n=3), carotid interventions (n=3), and other procedures (n=3). All had cardiovascular risk factors; 85% were on antithrombotic therapy. Branch retinal artery occlusion was most common (69.2%), followed by paracentral acute middle maculopathy (23.1%) and central retinal artery occlusion (7.7%). Symptom onset ranged from immediately post-procedure to 28 days, with varying clinical characteristics across different timeframes. Neuroimaging revealed cerebral ischemia in 7 of 10 patients (70%).

**Conclusions:**

Retinal ischemic events following cardiovascular procedures presented across a spectrum of timing, with varying clinical features that may suggest different pathophysiological mechanisms such as procedural embolization, perioperative hypoperfusion, or delayed complications from evolving embolic sources.The high rate of concurrent cerebral ischemia supports prompt brain imaging. These events, regardless of timing, should prompt comprehensive neurological and cardiological evaluation, as they may indicate systemic or evolving cardioembolic disease even in patients on antithrombotic therapy.

**Supplementary information:**

The online version contains supplementary material available at 10.1007/s00417-026-07143-1.

## Abstract (highlights)

**Key messages:**

**What is known:**

- Retinal ischemic events are rare but serious complications of cardiovascular procedures.

**What is new:**

- Most patients had substantial comorbidity, with 84.6% already receiving antithrombotic therapy before theirprocedures, yet ischemic events still occurred.
- Cerebral ischemia is commonly detected on neuroimaging after the ocular ischemic event, supporting consideration of brainimaging in this setting.
- Retinal embolic events may occur at varying times following cardiovascular procedures, potentially corresponding to differentischemic mechanisms.

**Supplementary information:**

The online version contains supplementary material available at 10.1007/s00417-026-07143-1.

## Introduction

Perioperative visual loss following non-ocular surgery is a rare but devastating complication, occurring in approximately 1 in 60,000 to 125,000 anesthetics, with highest rates reported after cardiac and vascular procedures [1]. Based on a large-scale national data, retinal artery occlusion occurs at a rate of 7.77 per 10,000 cardiac procedures [2].

Systemic vascular procedures pose significant risk for retinal ischemic complications through several well-described mechanisms [1–5]. Embolic events represent the predominant pathway, resulting from manipulation of atherosclerotic vessels and cardiac structures during the procedures. The ophthalmic artery, as the first major branch of the internal carotid artery, is particularly vulnerable to embolic material from cardiac chambers, aortic arch, and carotid atherosclerotic plaques [3, 5]. Hemodynamic instability and hypoperfusion represent an additional, non-embolic mechanism contributing to retinal ischemia [1, 4, 5].

Previous studies examining patients following cardiovascular procedures have focused primarily on screening for retinal emboli regardless of visual symptoms, typically examining patients at a single predetermined timepoint after procedure. Carotid interventions show retinal embolization rates of 4–5%, with symptomatic events occurring in approximately 1.7% of cases [6]. Studies of cardiac catheterization procedures have reported retinal emboli rates between 2% and 6.3%, with symptomatic events in approximately 0.3% of cases [7, 8]. Additionally, case reports have described symptomatic patients following various procedures, including abdominal surgeries [9].

This study aims to comprehensively characterize symptomatic retinal ischemic events across diverse systemic vascular procedures in a tertiary medical center over a 10-year period, with particular focus on timing of onset, clinical characteristics, and associated cerebrovascular findings.

## Methods

This retrospective study included patients aged 18 years and older diagnosed with acute retinal ischemic events at the Retina Service of the Goldschleger Eye Institute, Sheba Medical Center.

We systematically searched electronic medical records (between January 2015 to December 2024) using diagnostic terms including ‘retinal artery occlusion (RAO),’ ‘branch retinal artery occlusion (BRAO),’ ‘central retinal artery occlusion (CRAO),’ and ‘paracentral acute middle maculopathy (PAMM).’

Only patients with Optical Coherence Tomography (OCT)-confirmed diagnoses were selected, as OCT is essential for differentiating between these retinal ischemic conditions that can present with overlapping clinical features.

Records were reviewed to identify patients with acute ischemia occurring within 30 days following cardiovascular procedures. The 30-day timeframe was selected based on the standardized definition of perioperative stroke [10, 11]. For temporal analysis, patients were subsequently categorized into three groups: immediate onset (day 0), early onset (days 1–7), and delayed onset (days 8–30). This classification aligns with the American Heart Association framework distinguishing early postoperative strokes (≤ 7 days) from late postoperative strokes (> 7 days) [11]. We further separated same-day events to capture potential intraoperative embolization. Given the small sample size, this timing-based classification should be considered exploratory and hypothesis-generating. The systematic patient selection process is detailed in Supplementary Fig. 1. This screening yielded 13 patients for analysis, all with unilateral involvement.

All patients in the final cohort were hospitalized and underwent comprehensive systemic evaluation. De-identified data were collected on age, gender, medical history (systemic and ocular conditions), procedural details (from operative reports), post-event cardiovascular and neuroimaging findings, ophthalmic examination results, OCT findings, treatment details, and clinical outcomes.

Descriptive statistics were used to summarize patient demographics, clinical characteristics, and outcomes. Continuous variables are reported as mean ± standard deviation (SD), and categorical variables as counts and percentages. The study adhered to the tenets of the Declaration of Helsinki and was approved by the Institutional Review Board, which waived the requirement for informed consent owing to the retrospective design and anonymized use of existing clinical data.

## Results

### Study population and procedures

Thirteen patients (mean age 67 ± 8.5 years, 84.6% male) were identified with acute retinal ischemic events following cardiovascular procedures over the 10-year study period. Patient demographics and clinical characteristics are detailed in Table 1. All patients had cardiovascular risk factors, with the majority (77%) having multiple risk factors. Nearly half of the patients (46.2%) had established cardiovascular disease prior to the procedure. Most patients (84.6%) were already receiving antithrombotic therapy before their procedures.

**Table 1:** Patient Demographics, clinical Characteristics, and procedure types

Characteristic	*n* = 13	%
Demographics		
Age, mean ± SD (range)	67 ± 8.5 (51–79)	
Male sex	11	84.6
Cardiovascular Risk Factors		
Hypertension	11	84.6
Dyslipidemia	9	69.2
Diabetes mellitus	8	61.5
Atrial fibrillation	3	23
Smoking history	3	23
Prior Cardiovascular Disease		
Ischemic heart disease	6	46.2
Peripheral vascular disease	3	23
Prior cerebrovascular events	3	23
Prior Ocular History		
Pseudophakic	3	23
Prior laser refractive surgery	1	7.7
Prior retinal vascular disease	0	0
Pre-procedural Antithrombotic Therapy		
Any antithrombotic therapy	11	84.6
-Antiplatelet monotherapy	8	61.5
-Anticoagulation alone	1	7.7
-Combined therapy	2	15.4
No antithrombotic therapy	2	15.4
Procedure Category		
Total Cardiac Procedures	8	61.5
Cardiac catheterization	4	31
Aortic valve replacement	3	23
-TAVI	2	15.4
-Surgical AVR + CABG	1	7.7
Heart transplantation	1	7.7
Total Vascular Procedures	5	38.5
Carotid interventions	3	23
Peripheral vascular intervention	1	7.7
Aortic aneurysm repair	1	7.7

*SD * standard deviation, *TAVI * transcatheter aortic valve implantation, *CABG * coronary artery bypass grafting, *AVR * aortic valve replacement

The procedures included both cardiac (62%) and vascular interventions (38%). The most common procedures were cardiac catheterization (*n* = 4, 30.8%), valve replacement and carotid interventions (*n* = 3 each, 23.1%). All procedures were reported as uneventful. The only exception was the open abdominal aortic aneurysm repair in patient 7, which lasted 5 h and was associated with approximately 1.5 L of intraoperative blood loss. Procedure types are detailed in Table 1.

### Clinical spectrum and timing of retinal ischemic events following cardiovascular procedures

Individual patient details, including specific procedures, timing relationships, and clinical findings, are summarized in Tables 2 and 3.

**Table 2:** Summary of clinical findings by timing of onset

Characteristic	Immediate (Day 0, *n* = 4)	Early (Days 1–7, *n* = 5)	Delayed (Days 8–28, *n* = 4)
Diagnoses	BRAO (4/4, 100%)	BRAO (2), CRAO (1), PAMM (2)	BRAO (3), PAMM (1)
Visible emboli	2/4 (50%)	0/5 (0%)	4/4 (100%)
CWS/hemorrhages	0/4 (0%)	3/5 (60%)	1/4 (25%)
Preserved VA (≥ 20/40)	2/4 (50%)	3/5 (60%)	4/4 (100%)
Cerebral ischemia on imaging	2/3 imaged (67%)	3/4 imaged (75%)	2/3 imaged (67%)
Main procedures	Cardiac catheterization (3), Carotid stent (1)	Valve replacement (1), Vascular surgery (2), Cardiac catheterization (1), Carotid stent (1)	Valve replacement (2), Heart transplant (1), Carotid intervention (1)

*BRAO* branch retinal artery occlusion, *CRAO * central retinal artery occlusion, *CWS * cotton wool spots, *PAMM * paracentral acute middle maculopathy, *VA * visual acuity

**Table 3:** Detailed clinical characteristics, procedural data, and ophthalmologic findings of patients with post-procedural retinal ischemic events

Patient	Age	Sex	Procedure	Procedure Description	Time to Ocular Event	Retinal Ischemic Signs	Visible Emboli/CWS/Heme	Visual Acuity	Imaging Done	Imaging Significant Findings
1	51	M	Cardiac Catheterization	Stents to LAD+RPDA	Immediately	BRAO	No	20/25	CT, CTA, TTE	No visible infarcts
2	70	M	Carotid Catheterization	Right carotid stent placement	Immediately	BRAO	Emboli	20/1200	NA	NA
3	60	M	Cardiac Catheterization	Stent to LMCA and LCX	Immediately	BRAO	Emboli	CF	CT (brain and abdomen)	Brain CT- Acute fronto-cortical infarct and chronic infarcts. Abdomen CT- Multiple infarcts in the spleen and kidneys
4	76	M	Cardiac Catheterization	Stent to LAD	Immediately	BRAO	No	20/25	CT, TTE	Old lacunar infarcts
5	72	M	Leg Catheterization	Femoro-popliteal bypass stenosis	2 days	BRAO	CWS + hemorrhages	20/600	MRI, CTA	Chronic ischemia; Old infarcts in the cerebellum, Complete occlusion of the carotid artery
6	75	M	Valve Replacement	TAVI for severe aortic stenosis	2 days	BRAO	No	20/30	CT	Old lacunar infarcts
7	73	M	Abdominal Aortic Surgery	Open repair of aorto-bi-iliac aneurysm	3 days	PAMM	CWS + hemorrhages (Fig. 1A-C)	20/40	NA	NA
8	69	M	Cardiac Catheterization	Stent to LAD	7 days	CRAO	No	CF	CT, CTA, TTE	Suspected NPH, ICA stenosis without occlusion
9	62	F	Carotid Catheterization	Left carotid stent placement	6 days	PAMM	CWS + hemorrhages	20/20	MRI	No visible infarcts
10	60	M	Valve Replacement+ CABG	Aortic valve replacement + LIMA to LAD	12 days	BRAO	Emboli	20/30	TTE	NA
11	79	F	Valve Replacement	TAVI for severe aortic stenosis, followed by pacemaker implantation and ablation.	14 days	BRAO	Multiple Emboli (Fig. 2)	20/25	CT, TTE	CT- Atherosclerotic arterial changes without infarction. Echocardiography-Valvular vegetation detected
12	69	M	Cerebral and carotid Catheterization	Thrombectomy+ stent to RICA	19 days	PAMM	Multiple Emboli (Fig. 1D-F)	20/20	CT, TTE	Subacute infarct involving the basal ganglia
13	56	M	Heart Transplantation	Heart transplant	28 days	BRAO	Emboli + CWS	20/30	CT	Multiple acute cortical infarcts and an old chronic temporal infarct

*AF * Atrial Fibrillation, *BRAO * Branch Retinal Artery Occlusion, *CABG * Coronary Artery Bypass Grafting, *CF * Counting Fingers, *CT * Computed Tomography, *CTA * Computed Tomography Angiography, *CWS * Cotton Wool Spots, *Heme * Hemorrhages, *ICA* internal carotid artery, *LAD * Left Anterior Descending artery, *LCX * Left Circumflex artery, *LIMA * Left Internal Mammary Artery, *LMCA * Left Main Coronary Artery, *MRI * Magnetic Resonance Imaging, *NA * Not Available, *NPH* normal pressure hydrocephalus, *OCT * Optical Coherence Tomography, *PAMM * Paracentral Acute Middle Maculopathy, *RICA * Right Internal Carotid Artery, *RPDA * Right Posterior Descending Artery, *TAVI * Transcatheter Aortic Valve Implantation, *TTE * Transthoracic Echocardiography

BRAO was the predominant diagnosis, occurring in 9 patients (69.2%), CRAO occurred in 1 patient (7.7%) while isolated PAMM was identified in 3 patients (23%). Right eye involvement was observed in 8 patients (61.5%). Visible retinal emboli were identified in 6 patients (46.2%), cotton wool spots (CWS) in 4 patients (30.8%), and retinal hemorrhages in 3 patients (23%).

Visual acuity at presentation was preserved (20/20 to 20/40) in 9 patients (69.2%), while 4 patients (30.8%) had severe impairment (20/120 to counting fingers (CF).

Visual symptom onset demonstrated three temporal patterns:

Immediate presentation (day 0, *n* = 4, patients 1–4) occurred exclusively as BRAO, predominantly following cardiac catheterization (3/4). Visible emboli were identified in only 2 patients (patients 2 and 3), both of whom had severely impaired vision.

**Fig. 1:**
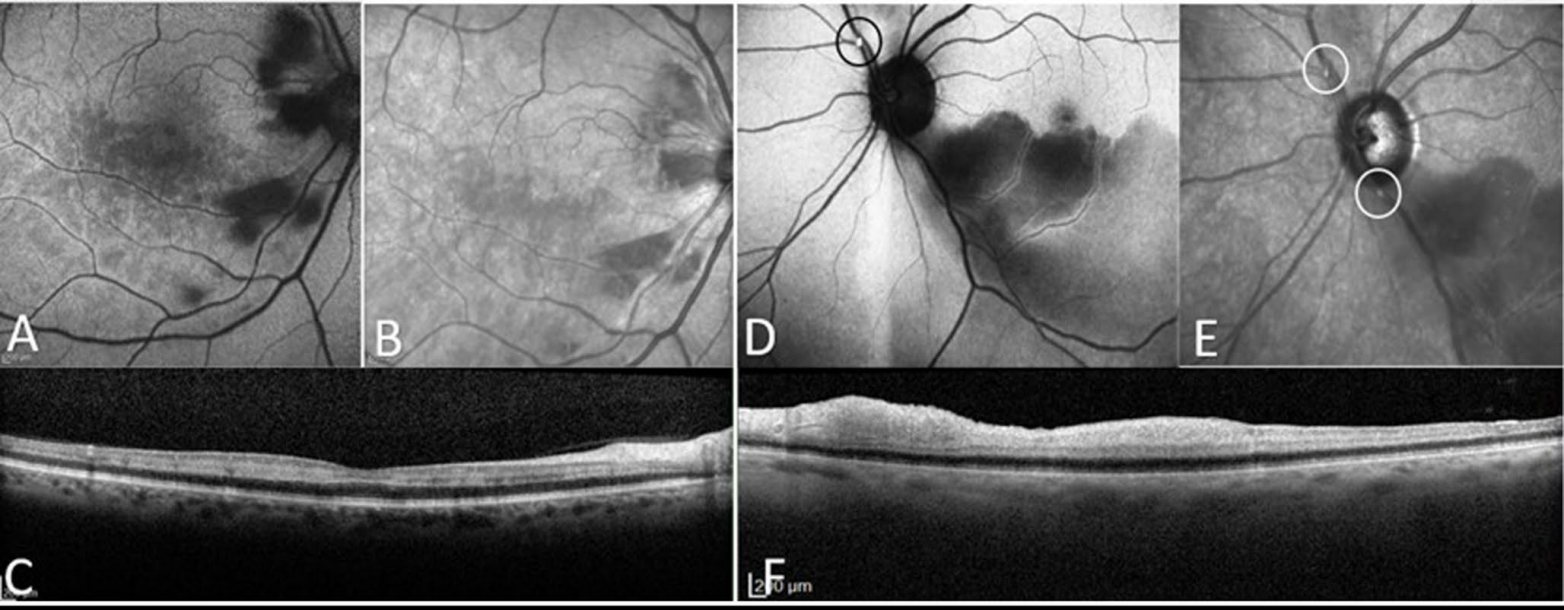
Multimodal imaging of Patient 7 and Patient 11. **A–C**: Patient 7, a 73-year-old male who presented with decreased vision three days after 5 h open surgical repair of an aorto-bi-iliac aneurysm. Best-corrected visual acuity (BCVA) was 20/40. (**A**) Fundus autofluorescence (FAF) image reveals cotton wool spots (CWS) and small superficial hemorrhage as well as perivenular paracentral acute middle maculopathy (PAMM). (**B**) Near-infrared reflectance (NIR) image shows corresponding lesions without identifiable retinal emboli. (**C**) A long optical coherence tomography (OCT) B scan demonstrates skipping hyperreflective lesions in the inner nuclear layer, consistent with PAMM. **D–F**: Patient 11, a 79-year-old female who developed decreased vision 14 days after undergoing transcatheter aortic valve implantation (TAVI), followed by pacemaker insertion and catheter ablation. BCVA was 20/25. (**D**) FAF image shows a hyperautofluorescent retinal embolus (circle) in the superior retina. (**E**) NIR image demonstrates two visible emboli (circle). (**F**) long OCT scan through the inferior retina reveals inner retinal hyperreflectivity consistent with acute branch retinal artery occlusion (BRAO)

Early presentation (days 1–7, *n* = 5, patients 5–9) included 2 BRAO, 1 CRAO and 2 isolated PAMM cases. No visible emboli were identified, yet 3 patients (patients 5, 7 and 8) demonstrated CWS and hemorrhages (Fig. 1A-C).

**Fig. 2:**
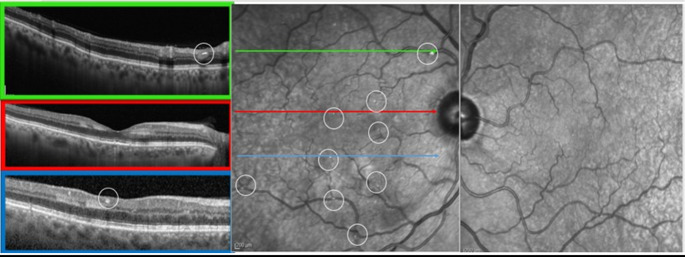
Multimodal imaging of Patient 12: A 69-year-old male presented with a new paracentral scotoma in the right eye 19 days after thrombectomy and stenting of the right internal carotid artery. Near-infrared reflectance (NIR) imaging shows multiple scattered retinal emboli (highlighted by a white circle).Three optical coherence tomography (OCT) B-scans are shown: The superior scan (green) demonstrates a hyperreflective embolus consistent with a plaque. The central scan (red) passes through the fovea and shows band-like hyperreflectivity in the middle retinal layers, consistent with paracentral acute middle maculopathy (PAMM). The inferior scan (blue) intersects a smaller embolus beneath the fovea and reveals additional PAMM lesions. Brain CT performed during the workup demonstrated a subacute infarct

Delayed presentation (days 8–28, *n* = 4, patients 10–13) comprised 3 BRAO and 1 PAMM (patient 11). All patients uniformly exhibited visible emboli, with 2 patients (10,11) showing multiple embolic events (Figs. 1D-F and 2). Despite visible emboli, visual acuity remained relatively preserved in this group. A summary of key clinical findings across timing groups is presented in Table 2.

### Systemic investigations and associated findings

Neuroimaging was performed in 10 patients (77%), echocardiography in 5 patients and abdominal Computed Tomography (CT) was performed in 1 patient. Imaging types and findings are detailed in Table 3. Cerebrovascular pathology was present in 8 of 10 patients imaged (80%), in 7/8 (87.5%) these findings were previously unknown, comprising chronic cerebral infarcts (*n* = 3), acute infarction (*n* = 2), subacute infarction (*n* = 1), and complete occlusion of the right internal carotid artery (*n* = 1). Neuroimaging was not performed in three patients, generally reflecting the admitting team’s decision in the absence of neurological symptoms, clinical stability and an initial perception of lower cerebrovascular risk.

Echocardiography revealed valvular vegetation in patient 11. Abdominal CT imaging demonstrated multiple embolic infarcts involving the spleen and kidneys in patient 3.

Following the retinal ischemic events, antithrombotic therapy was modified in 9 patients (69.2%): 2 patients initiated de novo treatment, 3 had an additional agent added, and 4 were switched to an alternative regimen. The remaining 4 patients (30.8%) continued their previous treatment without modification.

### Notable cases

Two patients experienced fatal outcomes, warranting detailed description due to the severity of their systemic embolic disease.

Patient 3 was a 60-year-old male with multiple cardiovascular risk factors who underwent cardiac catheterization with stent placement to the left main and left circumflex arteries. During the procedure, he developed immediate visual symptoms, presenting with BRAO, visible retinal emboli, and severely impaired vision (CF). Neuroimaging revealed acute fronto-cortical infarction alongside chronic cerebral infarcts. Due to new-onset renal dysfunction during follow up, abdominal CT was performed, revealing multiple embolic infarcts involving the spleen and kidneys, confirming widespread systemic embolization. The patient died during subsequent hospitalization due to complications of systemic embolic disease.

Patient 11 was a 79-year-old female who underwent transcatheter aortic valve implantation (TAVI) for severe aortic stenosis. Following the procedure, she developed atrial fibrillation with first-degree AV block and left bundle branch block, necessitating catheter ablation and dual-chamber pacemaker implantation. She developed visual symptoms 28 days after the TAVI and 14 days after pacemaker implantation, presenting with BRAO and multiple visible retinal emboli with preserved visual acuity (20/25). Echocardiography revealed valvular vegetation, with blood cultures positive for Cutibacterium avidum. She died less than 90 days from TAVI procedure as a result of complications related to infective endocarditis (Fig. 1D-F).

## Discussion

To our knowledge, this study represents the largest consecutive series of symptomatic retinal ischemic events following diverse systemic vascular procedures. Previous investigations have been limited to isolated case reports [12–16], or screening studies that detected mostly asymptomatic retinal emboli at predetermined time points after specific procedures [6–8]. These prior studies did not assess temporal evolution and retinal findings nor did they look at the correlation with neuroimaging results and complications.

Our cohort demonstrated a high burden of cardiovascular comorbidities and risk factors, with majority of patients having multiple risk factors. Half of the patients had established cardiovascular disease including IHD, peripheral vascular disease, or prior cerebrovascular events. These findings are consistent with the systemic associations of RAO described by Hayreh and colleagues [17]. Notably, most patients (84.6%) were already receiving antithrombotic therapy prior to their procedures, yet retinal ischemic events still occurred.

The timing variations observed in our series, raise the possibility of different underlying mechanisms. It is important to emphasize that this classification is exploratory and hypothesis-generating given the small sample size (*n* = 13, with 4–5 patients per group), and the proposed mechanisms represent hypotheses based on clinical observations rather than established pathophysiological distinctions. Immediate presentations likely reflect acute embolization during instrumentation, with emboli dislodged from the aortic arch or carotid arteries during catheter manipulation [6–8]. Visible retinal emboli were detected in only half of these cases, possibly due to emboli being below the detection threshold or the retrospective nature of the study without standardized fundus imaging.

The early group (2–7 days post-procedure) was characterized by the absence of visible emboli in all 5 cases, with CWS and hemorrhages seen in three patients, two of whom also had PAMM. In this group, two patients underwent prolonged or extensive procedures, including open abdominal aortic surgery lasting 5 h with significant blood loss and long peripheral bypass surgery. These features may suggest the involvement of hypoperfusion-related mechanism. For the remaining patients in the early-onset group, operative reports did not document specific intraoperative complications; however, given the retrospective design, detailed hemodynamic data were not consistently available.

In delayed presentations (8–30 days), visible retinal emboli were present in all four cases, with two patients showing multiple embolic events. Three patients in this group had undergone major cardiac procedures: two with valve replacement (one combined with CABG) and one with heart transplantation. This temporal pattern suggests delayed embolic events that may be originating from evolving sources such as thrombus formation on prosthetic valves or surgical suture lines, development of valve vegetations, or new-onset atrial fibrillation. However, patient 12, who underwent cerebral thrombectomy and carotid stenting, presented unique challenges in temporal classification due to his critical systemic condition, which may have precluded earlier reporting symptoms. These observations suggest that in cases of delayed-onset retinal ischemia following cardiovascular procedures, targeted evaluations may be considered, including echocardiography to assess for thrombus or valve vegetations and rhythm monitoring for new-onset atrial fibrillation.

The strong association between retinal ischemic events and concurrent cerebral ischemia in our cohort reinforces the shared vascular pathophysiology. Among our 10 patients who underwent neuroimaging, cerebrovascular pathology was identified in 8 (80%), of which 7 were previously undiagnosed. Three patients (33%) had acute or subacute cerebral infarction, aligning with meta-analytic data demonstrating concurrent acute cerebral ischemia in approximately 25–30% of patients with retinal artery occlusion [16]. Notably, most patients in our cohort underwent CT rather than MRI; given the lower sensitivity of CT for detecting acute ischemia, particularly early or small infarcts, the true rate of concurrent acute cerebral ischemia in our cohort may be underestimated [18]. These findings strongly support the American Heart Association recommendations for neurological evaluation and brain imaging in all patients presenting with retinal ischemic events, given the substantially increased stroke risk [19–21].

This study has several limitations. The small sample size of 13 patients limits statistical power and generalizability of findings. Importantly, while the temporal relationship between procedures and retinal ischemic events is compelling, causality cannot be definitively established; some events may have occurred coincidentally in patients with high baseline cardiovascular risk. Our case identification relied on documented diagnoses and OCT confirmation, potentially missing undiagnosed or unreported cases. The heterogeneous nature of procedures prevents procedure-specific risk assessment and limits our ability to determine which interventions carry the highest risk. Additionally, imaging relied on clinical examination and OCT without fluorescein angiography, potentially missing subtle and peripheral embolic events. Finally, delayed recognition of visual symptoms in critically ill patients may have affected the accuracy of temporal correlations between procedures and symptom onset.

In our series, retinal ischemic events presented across a spectrum from immediate to 28 days post-procedure, with varying clinical characteristics that may reflect different underlying mechanisms. These events occurred despite antithrombotic therapy in most patients and may serve as early indicators of systemic or evolving cardioembolic disease. The high rate of concurrent cerebral ischemia (70%) underscores the need for prompt neurological evaluation and brain imaging. Retinal ischemia following cardiovascular procedures, regardless of timing, should prompt comprehensive neurological and cardiological assessment, and multidisciplinary collaboration among ophthalmology, cardiology, and neurology is essential for optimal patient management. Larger studies are needed to confirm these patterns and inform preventive strategies.

## Supplementary Information

Below is the link to the electronic supplementary material.

Supplementary Material 1 (DOCX 122 KB)

## Data Availability

De-identified data underlying this article are available from the corresponding author on reasonable request, subject to institutional policies and patient privacy constraints.

## Declarations

### Clinical trial registration

Clinical trial number: not applicable.
