# Supplementary material for: Retinal ischemia after cardiovascular interventions: Neuroimaging correlates and timing phenotypes in a consecutive series
Source: Graefes Arch Clin Exp Ophthalmol. 2026 Feb 24;264(6):1795–803. doi: 10.1007/s00417-026-07143-1 (PMC13197270; doi:10.1007/s00417-026-07143-1)
Supplement: Supplementary file 1 [file 417_2026_7143_MOESM1_ESM.docx]

Supplementary Material

Supplementary Figure 1.

Figure 1. Patient selection flowchart. From 799 retinal ischemic cases identified through systematic database search, 368 acute cases with complete records were identified. Following OCT confirmation (n=248) and screening for cardiovascular procedures within 30 days, 13 patients comprised the final study cohort.

Abbreviations: RAO, retinal artery occlusion; BRAO, branch retinal artery occlusion; CRAO, central retinal artery occlusion; PAMM, paracentral acute middle maculopathy; OCT, optical coherence tomography;
